# Seropositivity and Risk Factors for Herpes Simplex Virus Type 2 Infection among Female Sex Workers in Guangxi, China

**DOI:** 10.1371/journal.pone.0069697

**Published:** 2013-07-22

**Authors:** Shaochun Chen, Yueping Yin, Xiangsheng Chen, Hongchun Wang, Yanhua Yu, Wanhui Wei, Yan Han, Ning Jiang, Baoxi Wang

**Affiliations:** National Center for STD Control, and the Chinese Academy of Medical Sciences and Peking Union Medical College Institute of Dermatology, Nanjing, China; Tulane University, United States of America

## Abstract

**Objectives:**

To determine seropositivity of herpes simplex virus type 2 (HSV-2) infection and associated risk factors among female sex workers (FSWs) in Guangxi, China.

**Methods:**

A convenience sample of FSWs was recruited from different types of sex work venues in two cities (Wuzhou and Hezhou) in Guangxi. Blood specimens were collected for ELISA-based detection of HSV-2 antibodies to examine the seropositivity of HSV-2 infection. Socio-demographic and behavioral data were collected through a structured questionnaire interview. Association of HSV-2 seropositivity with socio-demographic and behavioral characteristics and HIV status was analyzed.

**Results:**

The overall prevalence of HSV-2 seropositivity among 2453 FSWs was 54.9% (95% confidence interval [CI], 52.9–56.9%). The HSV-2 seropositivity was independently associated with older age, low education level, non-Han minority, migration status, working in lower-tier venues and positive HIV status.

**Conclusions:**

The study indicates a high prevalence of HSV-2 infection among FSWs, particularly in those working in low-tier venues in study areas, suggesting the needs to further emphasize the inclusion of HSV-2 in surveillance and intervention programs in this population.

## Introduction

Herpes simplex virus type 2 (HSV-2) infections is one of the most prevalent sexually transmitted infections (STIs) in the world and is a major cause of genital ulcers [1]. An increasing HSV-2 prevalence has been found throughout the world recent years especially among high risk population. Some epidemiologic and clinical studies suggest strong multiple interactions between HSV-2 and human immunodeficiency virus (HIV) [2], [3]. HSV-2 was also identified as a risk factor for HIV infection in some epidemiological studies and meta-analyses [4], [5]. In addition, HSV-2 antibody status can be used as a serologic marker for HIV vulnerability for surveillance and intervention purposes [6].

China's HIV and STI epidemic varies widely across the country. Six provinces with the most reported cases, including Guangxi are classified as high prevalence areas, and account for 70–80% of total reports nationwide [7]. Guangxi was also one of the three provinces with highest burden of syphilis and gonorrhoea in China according to the reports from the national STI surveillance system in 2012 [8]. However, data concerning common STIs, including HSV-2 infections among most-at-risk populations are limited. Female sex workers (FSW) are one of the important populations to drive the local HIV/STI epidemic and are thought to act as the bridge population for the heterosexual transmission of HIV/STIs from high-risk groups to the general population [9]. Therefore, timely monitoring of other STIs, particularly those that can be used as proxies of risk behaviors in the population, will be helpful for developing efficient surveillance and intervention programs. The current study aimed to determine the seroprevalence of HSV-2 infection in a sample of FSWs recruited from two cities in Guangxi, and to identify significant factors associated with this infection.

## Materials and Methods

### Ethics Statement

The study protocol was reviewed and approved by the Medical Ethics Committee at Institute of Dermatology, the Chinese Academy of Medical Sciences & Peking Union Medical College and National Center for Sexually Transmitted Disease Control, Nanjing, China. Written informed consent was obtained from all the participants or the caretakers of the minors/children participants involved in our study.

### Study Design and Participants

The current study was a cross-sectional study to recruit a convenience sample from different types of sex work venues in two cities (Wuzhou and Hezhou) in Guangxi Zhuang Autonomous Region from June to September, 2009. Sex work venues were mapped to determine the nature and location and classified into three types (high-, middle- and low-tier venues) of venues. FSWs were categorized into three subgroups based on the sex work venues where FSWs solicited clients. High-tier FSW were those who solicited clients from karaoke bars, or hotels; middle-tier FSWs were those from hair salons or barber shops, massage parlors, foot bathing shops, roadside shops, guesthouses, or roadside restaurants; and low-tier FSWs were those on the street or in public outdoor places.

FSWs were sampled from each of the three categories, with oversampling from middle- and low-tier venues. Participants eligible for this study were females who were older than 15 years and who reported having commercial sex with clients in the past three months, and gave an informed consent. This survey was integrated into the local routine outreach services in the study sites, and the study details have been reported elsewhere [9]. Briefly, after the venues to be investigated were selected, an outreach team consisting of a public health workers and technicians went to explain the study description and purpose, and went through the individual informed consent process. An informed consent was obtained from the eligible participant before the structured questionnaire interview was conducted and blood specimens were collected according to study protocols. In order to keep the questionnaire survey as simple as possible, a few questions relevant to risk behaviors (e.g., condom use in the most recent sex with sexual partner, frequency of condom use in the last month, and any illicit drug use in the past year) were included in addition to socio-demographic information.

### Laboratory Assays for Detecting HSV-2 and HIV Seropositivity

Sera were separated from the blood samples and stored at -70°C prior to the laboratory testing. Enzyme-linked immunosorbent assay (ELISA) (Captia HSV 2 type specific IgG; Trinity Biotech, Jamestown, NY, USA) was used to detect IgG antibodies to HSV-2. Sera were screened for HIV antibodies by ELISA (Anti-HIV1/2 EIA Kit; Livzon Group Reagent Factory, Zhuhai, Guangdong, China) and a western blot (Singapore MP Biomedical Asia Pacific Ltd, Singapore) was used to confirm initially reactive ELISA results. HIV infection was defined as positivity to both ELISA and western blot, and HSV-2 seropositivity was defined as having detectable IgG antibodies to HSV-2. For those specimens with ambiguous test results, a repeat test was performed to determine the positivity. If the result was still ambiguous, the sample was considered to be negative. The HIV testing was conducted locally with quality control from the National STD Reference Laboratory and the HSV-2 testing was conducted in the Reference Laboratory.

### Statistical Analyses

All participants who answered the questionnaire survey and provided blood specimens were included into final analysis. All data from the questionnaires and laboratory tests were double entered into a computer using EpiData software (EpiData Association, Denmark) by two research assistants. The univariate analysis was performed by using Chi-square (χ^2^) test to determine the association of HSV-2 infection with individual socio-demographic and behavioral factors. The variables that were significant at the level of P<0.05 in the univariate analysis were included in the multivariate logistic regression model to evaluate the factors associated with the infection and to estimate adjusted odds ratio (AOR) and 95% confidence intervals (95%CI) for each factor. Variables reaching p<0.05 was considered statistically significant. SPSS version 13.0 (SPSS Inc, USA) was used for the statistical analysis.

## Results

### Socio-Demographic and Behavioral Characteristics of FSWs

The demographic characteristics of our study population are presented in table 1. A total of 2,453 FSWs, consisting of 1,115 (45.5%) FSWs from high-tier venues, 993 (40.5%) from middle-tier venues and 225 (9.2%) from low-tier venues, were enrolled from Wuzhou City (n = 1097, 44.7%) and Hezhou City (n = 1356, 55.3%). The mean and the median age were 26.7 years (standard deviation [SD] ±7.1, range from 16 to 55 years) and 25 years (interquartile range [IQR] = 21–31 years), respectively. Majority of the participants (81.7%) were of Han ethnicity. About one-quarter of the participants (555, 22.6%) had education level of primary school or below, and more than 60% (1547, 63.1%) received an education at junior high school. More than half of participants (n = 1364, 55.6%) were unmarried. About half of the participants were local registered residents (n = 1151, 46.9%), and another half migrated from other cities in Guangxi (624, 25.4%) or from other provinces in China (675, 27.5%).Most of FSWs (n = 1942, 79.2%) reported using a condom during last sex and half of them reported consistent condom use during the last month (1365, 55.7%). Only 159 (6.5%) FSWs reported drug use in the previous year.

**Table 1:** Seropositivity and factors associated with HSV-2 infection.

Variable	Number		HSV-2 infection						
			Number	Seropositivity %(95% CI)		OR (95%CI)	P value	AOR (95% CI)	P value
**Total**	2453		1347	54.9 (52.9–56.9)					
**Recruitment region**									
Wuzhou	1097		575	52.4 (49.5–55.4)		1 (Reference)			
Hezhou	1356		772	56.9 (54.3–59.5)		1.20 (1.02–1.41)	0.025*		
**Age in years**									
≤26	1408		666	47.3 (44.7–49.9)		1 (Reference)		1 (Reference)	
>26	1045		681	65.2 (62.2–68.0)		2.08(1.77–2.46)	<0.001*	1.96 (1.55–2.47)	<0.001
**Ethnic group**									
Han	2004		1066	53.2 (51.0–55.4)		1 (Reference)		1 (Reference)	
Non–Han minority	449		281	62.6 (58.0–66.9)		1.47 (1.19–1.82)	<0.001*	1.34 (1.06–1.70)	0.016
**Educational level**									
Primary school orbelow	555		385	69.4 (65.4–73.1)		1 (Reference)		1 (Reference)	
Junior high school	1547		799	51.6 (49.2–54.1)		0.47 (0.38–0.58)	<0.001*	0.60 (0.47–0.75)	<0.001
Senior high schoolor above	351		163	46.4 (41.3–51.7)		0.38 (0.29–0.51)	<0.001*	0.50 (0.36–0.68)	<0.001
**Marital status**									
Never Married	1364		676	49.6 (46.9-52.2)		1 (Reference)		1 (Reference)	
Ever Married	1089		671	61.6 (58.7–64.5)		1.63 (1.39–1.92)	<0.001*	1.08 (0.84–1.40)	0.563
**Registered residence**									
Sampling city	1151		506	44.0 (41.1–46.8)		1 (Reference)		1 (Reference)	
Different cities	624		411	65.9 (62.1–69.5)		2.46 (2.00–3.01)	<0.001*	2.61 (2.07–3.29)	<0.001
Different provinces	675		427	63.3 (59.6–66.8)		2.20 (1.81–2.67)	<0.001*	1.80 (1.44–2.25)	<0.001
**Condom use during last sexual intercourse**									
Yes	1942		1113	57.3 (55.1–59.5)		1 (Reference)		1 (Reference)	
No	422		198	46.9 (42.2–51.7)		0.66 (0.53–0.81)	<0.001*	1.00 (0.74–1.36)	0.995
**Condom use frequency during the last month**									
Never	136		52	38.2 (30.5–46.6)		1 (Reference)		1 (Reference)	
Sometimes	853		459	53.8 (50.5–57.1)		1.88 (1.30–2.73)	0.001*	1.33 (0.85–2.10)	0.234
Every times	1365		793	58.1 (55.5–60.7)		2.24 (1.56–3.22)	<0.001*	1.49 (0.90–2.46)	0.126
**Drug use**									
Yes	159		94	59.1 (51.4–66.5)		1 (Reference)			
No	2294		1253	54.6 (52.6–56.7)		1.20 (0.87–1.67)	0.271		
**Working venue tier**									
High	1115		536	48.1 (45.2–51.0)		1 (Reference)		1 (Reference)	
Middle	993		605	60.9 (57.9–63.9)		1.68 (1.42–2.00)	<0.001*	1.47 (1.20–1.79)	<0.001
Low	225		161	71.6 (65.3–77.1)		2.72 (1.99–3.71)	<0.001*	1.76 (1.23–2.51)	0.003
**HIV infection**									
Negative		2437	1	0.04 (0.01–0.23)	1 (Reference)			1 (Reference)	
Positive		16	15	93.8 (71.7–98.9)	12.44(1.64–94.35)		0.015*	12.58 (1.58–100.54)	0.017

OR = odds ratio; CI = confidential interval; AOR = adjusted odds ratio.

* P<0.05 for chi-square (χ^2^) test.

### Seropositivity of HSV-2

Out of 2,453 FSWs, 1347 were HSV-2 seropositive, giving an overall seropositivity prevalence of 54.9% (95% CI, 52.9–56.9%). As shown in Table 1, the HSV-2 seropositivity prevalence was different in these two areas, being 56.9% (772/1356) in Hezhou and 52.4% (575/1097) in Wuzhou (P = 0.025). Compared with their counterparts, the HSV-2 prevalence was higher among FSWs aged 26 years or older (65.2%; P<0.001), being non-Han (62.6%; P<0.001), having less education level (69.4%; P<0.001), married status (61.6%; P<0.001) or a HIV positive status (93.8%; P = 0.015). FSWs who were locally registered residents had a lower prevalence (44.0%) than those who had migrated from other cities in Guangxi (65.9%; P<0.001) or other provinces in the country (63.3%; P<0.001). Condom use during the last sex or during the last month was associated with the HSV-2 seropositivity (P<0.001). HSV-2 positivity was higher among low-tier FSWs (71.6%), compared to middle-tier FSWs (60.9%; P = <0.001) and high-tier FSWs (48.1%; P<0.001).

### Risk Factors Associated to HSV-2 Seropositivity

In the multivariate logistic model (Table 1), HSV-2 infection was independently associated with the following factors: age of 26 years or older (AOR = 1.96, 95% CI, 1.55–2.47, P<0.001), low education level (AOR = 0.60, 95% CI, 0.47–0.75 for Junior high school level and AOR = 0.50, 95% CI, 0.36–0.68 for senior school or above level; both p<0.001), non-Han minority (AOR = 1.34, 95% CI, 1.06–1.70, P = 0.016), migration from other cities (AOR = 2.61, 95% CI, 2.07–3.29, P<0.001) or other provinces (AOR = 1.80, 95% CI, 1.44–2.25, P<0.001), working in lower-tier venues (AOR = 1.76, 95% CI, 1.23–2.51, P = 0.003) and positive HIV status (AOR = 12.58, 95% CI, 1.58–100.54, P = 0.017).

## Discussion

Several different studies conducted in different parts of China have reported varying HSV-2 seropositivity among FSWs in China (Table 2) [10], [11], [12], [13], [14], [15]. The overall prevalence of HSV-2 seropositivity in this study was 54.9% (1347/2453), which is slightly lower than that in three studies conducted in Yunnan province (65.1% in 2000 [10], 68% in 2006 [12] and 70.8% in 2006 [14]). A study in Shanghai in 2009 showed a similar prevalence (47.3%) [15]. In addition to the reflection to geographic difference in the epidemic, the differences among these studies could be attributed to socio-economic, cultural and behavioral nature of the study population, sampling strategies used, and sample size.

**Table 2:** Comparison among different studies on HSV-2 seropositivity in FSWs in China.

Study	study time	Region	Province	Sites and venues	Sample size	HSV-2 prevalence
(Wei, Chen et al. 2004) [13]	1999	Central China	Hubei	sauna/massage parlors, dancing/karaoke bars and barbershops in Wuhan	147	29.7%
(Chen, Yin et al. 2005) [10]	2000	Southwestern China	Yunnan	local reeducation center or STD clinics in Kunming	505	65.1%
(Ngo, Laeyendecker et al. 2008) [11]	2004	Southwestern China	Yunnan	local reeducation center or STD clinics in Kunming (including male sex workers)	500	33.0%
(Wang, Wang et al. 2008) [12]	2006	Southwestern China	Yunnan	FSWs in Kaiyuan City	737	68.0%
(Xu, Wang et al. 2008) [14]	2006	Southwestern China	Yunnan	entertainment venues (e.g., KTV, nightclubs, and hair salons) in Gejiu City	96	70.8%
(Yang, Yao et al. 2011) [15]	2008–2009	Eastern China	Shanghai	entertainment establishment venues in Shanghai	793	47.3%
(This study)	2009	Southern China	Guangxi	different tier venues in Guangxi Zhuang Autonomous Region	2453	54.9%

We found that HSV-2 prevalence varied among FSWs from different tiers of sex work venues, probably indicating different levels of risk in their sexual behaviors. FSWs working in low-tier venues tend to offer a lower cost service, but have a greater number of clients [16]. They have been found to be less likely to use a condom during commercial sex to meet the needs of clients [17]. The high prevalence of HSV-2 infection in this subgroup of FSW further calls for innovative action to address this particular population who are often neglected by traditional interventional programs.

The findings in our study show a significant association between HSV-2 infection and older age and this is consistent with that reported in previous study [15]. Such association may be explained by the fact that older FSWs have often had a longer career in sex work and consequently have had a long exposure to the risk of infection. Migration status was associated with HSV-2 infection. This association is consistent with a previous study in which rural-to-urban migrants were found to have higher sexual risks and poor STIs prevention knowledge than local residents [11], [18].

Consistent use of condoms has been reported to be associated with lower rates of infection with HSV-2 in previous studies [19], [20], [21]. Surprisingly, our study indicated an association of HSV-2 infection with more consistent condom use. Although more studies are needed to clarify this phenomenon in the particular population, recall and/or reporting bias may be one of the important concerns. Similar to the previous studies [3], HSV-2 infection was found to be associated with HIV infection. Such synergistic interactions of these infections further reveal the predominant sexual transmission of HIV and further emphasize the importance of preventing HSV-2 infection in this population.

The current study has some strengths, including recruitment of FSWs from different categories of sex work venues. However, some limitations in the study should be addressed. First, a convenience sampling rather than a random sampling was used to recruit participants from each venues may result in the sample bias and possible poor representativeness. Second, reporting and recall bias may have occurred during the questionnaire interview as it was based on self reported information. Third, although the current study investigated a few of common risk behaviors to be possibly related to the infection, more information about the risk behaviors, including the number of sexual partners, frequency of sexual intercourse, duration of commercial sex work, and sexual debut time were not included, and this has limited the exploration of relationship between the infections with potential risk factors. These limitations should be addressed in the further studies in the future.

### Conclusions

It can be concluded from the study that prevalence of HSV-2 seropositivity is high among FSWs, particularly those women working in low-tier sex work venues. In addition, HSV-2 seropositivity was also found to be associated with HIV infection and some socio-demographic characteristics. These findings will be helpful for designing the STI surveillance programs and developing the innovative interventions among FSW population in China.
